# Supplementary material for: Identifying indicators influencing emergency department performance during a medical surge: A consensus-based modified fuzzy Delphi approach
Source: PLoS One. 2022 Apr 21;17(4):e0265101. doi: 10.1371/journal.pone.0265101 (PMC9022798; doi:10.1371/journal.pone.0265101)
Supplement: S1 File — (DOCX) [file pone.0265101.s001.docx]

**Supplementary Materials**

**S1 Table. Pros & Cons of all the Methods**

| Method | Pros | Cons |
| --- | --- | --- |
| Non-analytical methods | 1. Easiness of gathering a mix set of professionals. 2. Wide circulation of results via the media and other outlets. 3. Unbiased panel. | 1. No structured interaction during the panel sessions. 2. Implicitly of the aggregation methodology 3. Lack of analytical/statistical methods for aggregation and consistency check. 4. Frequent disagreements during the sessions. |
| Nominal group technique | 1. Respondents meet face to face 2. Respondents have the opportunity to voice their opinions on each item. 3. Personal contact and interaction between the respondents. 4. Group voting can occur if desired in later rounds. | 1. Certain respondents in the session can take over discussion and drive results. 2. Time and economically expensive. 3. Limited solution to a few problems restricting its applicability to multiple scenarios. |
| Delphi method | 1. Avoiding direct confrontations 2. Enabling group communication free from geographical constraints. 3. Enabling anonymity 4. Reducing the noise 5. Enriching expert insight via feedback 6. Saving money and time; | 1. Lack of generalizability of the findings. 2. Obliviousness to reliability measurement. 3. Potential for bias 4. Coordinating large groups in several rounds 5. Lack of guidance and agreed standards 6. Low response rate 7. Misinterpret expert opinion. 8. No addressing the fuzziness of opinions |
| Fuzzy Delphi method | 1. Less number of surveys required. 2. Lower cost and less timely. 3. Simple calculation process 4. Completeness and consistency of the group opinion. 5. True reflection of experts’ response. | 1. Distortion of experts' opinions when represented by fuzzy numbers. 2. Loss of information when only distance measure (such as Euclidean distance) is used. |

*Delphi method (DM)*

The Delphi method (DM) is used due to its iterative consultation process with experts to communicate their opinions and knowledge about a complex problem. The method can be explained in four steps, as given below [1].

1. Analysis of responses: The standard descriptive statistical analysis which includes the mean, median, standard deviation, and interquartile range (IQR) is used to analyze the expert responses. A contingency table is created with the results.
2. Determine the consensus level: The median score is used to describe the importance rating of the measures, while a decision was made to calculate the acceptable degree of proximity and stability in the answers obtained during the rounds through the calculation of the IQR. The analysis of the consensus reached between experts’ opinions showed an acceptable degree of proximity and stability (IQR < 0.5) [1]. See S2 Table for the establishment of consensus criteria among experts

| **S2 Table. Consensus definition for the Delphi panel** | |
| --- | --- |
| Consensus | Parameters (Mdn: Median; IQR: Interquartile range) |
| Agreement (A) | Mdn $\geq$ 4, IQR $\leq$ 1.5 |
|  | Mdn $\geq$ 4, IQR $\leq$ 2, frequency [4-5] $\geq$ 70% |
| Disagreement (D) | Mdn $\leq$ 3.5, IQR $\leq$ 1.5 |
|  | Mdn $\leq$ 3.5, IQR $\leq$ 2, frequency [1-3] $\geq$ 70% |
| Neutral (N) | Mdn $\geq$ 3.5, IQR $\leq$ 2 |

1. Share feedback with experts: After each round, feedback is shared with experts so they can view the group responses and make modifications in the new round.
2. Reiterate until consensus is reached: If consensus is not reached in the previous round, the questionnaire is revised, and a new round created for experts to review and complete the items.

*Fuzzy Delphi method*

Applying the fuzzy Delphi method (FDM) to group decision can solve the fuzziness of common understanding of expert opinions. This study applied the triangular membership functions and the fuzzy theory to solving the group decision. The fuzziness of common understanding of experts could be solved by using the fuzzy theory and evaluated on a more flexible scale. The FDM steps are as follows [2-4]:

1. Collect opinions of decision group: Find the evaluation score of each indicator’s significance given by each expert by using linguistic variables in questionnaires.
2. Set up triangular fuzzy numbers: Convert the linguistic variables into triangular fuzzy numbers (see S3 Table). Let the fuzzy numbers $\tilde{r}_{ij}^{k}$ be the rating of factors $i$ w.r.t indicators $j$ and $\tilde{w}_{j}^{k}$ be the $jth$ indicator weight of the $kth$ expert for $i=1,\ldots,m;j=1,\ldots,n;k=1,\ldots K$ and $\tilde{r}_{ij}=\frac{1}{k}\left[ \tilde{r}_{ij}^{1}\oplus\tilde{r}_{ij}^{2}\oplus\ldots\oplus\tilde{r}_{ij}^{K} \right]; \tilde{w}_{j}=\frac{1}{k}\left[ \tilde{w}_{j}^{1}\oplus\tilde{w}_{j}^{2}\oplus\ldots\oplus\tilde{w}_{j}^{K} \right]$. The operation laws for two triangular fuzzy numbers $\tilde{m}=(m_{1},m_{2},m_{3})$ and $\tilde{n}=(n_{1},n_{2},n_{3})$ is given as:

$$\tilde{m}+\tilde{n}=\left( m_{1}+n_{1},m_{2}+n_{2}, m_{3}+n_{3} \right),$$

$$\tilde{m}\otimes\tilde{n}=\left( m_{1}n_{1},m_{2}n_{2},m_{3}n_{3} \right),$$

$$a\otimes\tilde{m}=\left( am_{1},am_{2}, am_{3} \right),$$

$$a>0$$

| **S3 Table. Linguistic variable to triangular fuzzy numbers** | |
| --- | --- |
| Variable | Fuzzy Scale |
| Strongly disagree | (0.0, 0.1, 0.2) |
| Disagree | (0.1, 0.2, 0.4) |
| Neutral | (0.2, 0.4, 0.6) |
| Agree | (0.4, 0.6, 0.8) |
| Strongly agree | (0.6, 0.8, 1.0) |

1. Compute the distance: For each medical professional, we use the weighted Euclidean distance to compute the distance between the average $\tilde{r}_{ij}$ and $\tilde{r}_{ij}^{K}$ and the distance between the average $\tilde{w}_{j}$ and $\tilde{w}_{j}^{k}, k=1,\ldots K$. The distance between two fuzzy numbers $\tilde{m}=\left( m_{1},m_{2},m_{3} \right)$ and $\tilde{n}=\left( n_{1}, n_{2}, n_{3} \right)$ is computed by

$d\left( \tilde{m}, \tilde{n} \right)=\sqrt{\frac{\sum_{i=1}^{n} \left( \tilde{m}_{i}-\tilde{n}_{i} \right)^{2}}{3}}$ (1)

1. Set a threshold: A threshold value is set to check for consensus if the medical professional’s evaluation data is less than the threshold value of 0.2 or the percentage of achieving a group consensus is greater than 60%, then we go to step 5; otherwise, a new round of survey is required.
2. Aggregate fuzzy assessment: We collect and aggregate each medical professional opinion to obtain a group opinion using equation 2 below:

$$\tilde{A}=\left[ \begin{matrix} \tilde{A}_{1} \\ \tilde{A}_{2} \\ \begin{aligned} \vdots\\ \tilde{A}_{m} \end{aligned} \end{matrix} \right] (2)$$

$$where \tilde{A}_{i}=\tilde{r}_{i1}\otimes\tilde{w}_{1}\oplus\tilde{r}_{i2}\otimes\tilde{w}_{2}\oplus\ldots\oplus\tilde{r}_{in}\otimes\tilde{w}_{n},i=1,\ldots,m$$

3. Defuzzification: The center of gravity method is applied to Defuzzify the fuzzy weight of each factor. $\tilde{A}_{i}=\left( a_{i1},a_{i2},a_{i3} \right)$ is defuzzified by

$a_{i}=\frac{1}{3}\left( a_{i1},a_{i2},a_{i3} \right)$ (3)

The ranking order of alternative options can be determined according to the values of $a_{i}$.

Our Proposed Modified Fuzzy Delphi

The approach consists of nine steps which is summarized in the mathematical expression below. Suppose that $O=\left\{ O_{n} | n=\bar{1,N} \right\}$ is a set of *N* healthcare performance factors, where each factor represents a finite set of indicators (i.e., *P_n_*), $I_{n}=\left\{ I_{np} | p=\bar{1,P_{n}} \right\} \forall O_{n}\in O$. We invite a group of healthcare experts, *E* of size *k* to analyze each indicator, $E_{np}=\left\{ E_{npk} | k=\bar{1,K_{np}} \right\} \forall I_{np}\in I_{n}$. Based on their experience and knowledge, each expert uses the Likert five-level scale to make a judgment on the given questionnaire survey. Since the experts remain anonymous (i.e., there is no physical information exchange among them), a random distribution of the responses for each indicator is the most probable. Their responses are based on the Likert five-level scale which is represented as a set of triangular fuzzy number on the interval [0 – 1] (see S1 Fig). We collect each individual panel member opinion and then aggregate the opinions to obtain a group opinion and a level of agreement (i.e., a threshold) for each indicator is obtained. Any indicator that exceeds the threshold is accepted while those that is below the threshold is revised and a new round conducted.

1. Converting each expert’s opinion into triangular fuzzy numbers: The experts complete the designed questionnaire based on a 5-point Likert scale. To address the subjectivisms of their responses, we will represent each expert's opinion (i.e., linguistic variable) using triangular fuzzy numbers (see S3 Table).
2. Computing the similarity $S_{w}(\tilde{R}_{i},\tilde{R}_{j})$ between each pair of experts’ opinions $\tilde{R}_{i}=a_{i}, b_{i}, c_{i}$ and $\tilde{R}_{j}=a_{j}, b_{j}, c_{j}$ for the *n* number of healthcare experts with *i* and *j* responses (i.e., linguistic indicators) [5] by:

$$S_{w}\left( \tilde{R}_{i},\tilde{R}_{j} \right)=\frac{\int_{x} \left( \min\left\{ \mu_{\tilde{R}_{i}}\left( x \right),\mu_{\tilde{R}_{j}}\left( x \right) \right\} \right)^{2}dx}{\int_{x} \left( \max\left\{ \mu_{\tilde{R}_{i}}\left( x \right),\mu_{\tilde{R}_{j}}\left( x \right) \right\} \right)^{2}dx} (4)$$

where the triangular fuzzy set $R_{i}$ having membership function $\mu_{\tilde{R}_{i}}\left( x \right)$ [6] is given by:

$\mu_{\tilde{R}_{i}}\left( x \right)=\left\{ \begin{aligned} 0 if x<\gamma-\alpha, \gamma+\beta\leq x, \\ \frac{x-\gamma+\alpha}{\alpha} if \gamma-\alpha\leq x<\gamma, \\ \frac{\gamma+\beta-x}{\beta} if \gamma\leq x<\gamma+\beta, \\ 0 if x>\gamma+\beta\end{aligned} \right.$ (5)

where $\gamma- \alpha=a_{i}; \gamma=b_{i}; \gamma+\beta=c_{i}$. S1 Fig depicts the representation of expert opinions as a triangular fuzzy number where the *x-axis* is the fuzzy set, and the *y-axis* is the corresponding membership function.

**S1 Fig. A triangular fuzzy number**

1. Computing the distance $d(\tilde{R}_{i},\tilde{R}_{j}$*)* between each pair of experts ${(\tilde{R}}_{i})\mathrm{and}(\tilde{R}_{j})$. Distance is an important concept in fuzzy set theory and a significant index when comparing fuzzy numbers [7]. Many studies have proposed different distance measures, and for our study, we will adopt the weighted Hamming distance measure and apply it to compute the distance between each pair of medical professional responses. According to Lu, Lan (5) the Hamming distance for any two fuzzy numbers *A* and *B* with membership functions $\mu_{\tilde{A}}$ and $\mu_{\tilde{B}}$, respectively, is represented as:

$$d_{H}\left( \tilde{A}, \tilde{B} \right)=\int_{x} |\mu_{\tilde{A}}\left( x \right)-\mu_{\tilde{B}}\left( x \right)|dx (6)$$

For the fuzzy numbers $\tilde{R}_{i}=(a_{i}, b_{i}, c_{i})$ $(i=1, 2,\ldots,n)$ of each expert’s opinion, the distance $D(\tilde{R}_{i},\tilde{R}_{j}$)

between each pair of $\tilde{R}_{i}\mathrm{and}\tilde{R}_{j}$ is measured. Then each distance is divided by $D^{*}\left( \tilde{R}_{p}, \tilde{R}_{q} \right)$ $\max_{i,j} D\left( \tilde{R}_{i}, \tilde{R}_{j} \right)$ to obtain the normalized distance in equation 7:

$d\left( \tilde{R}_{i}, \tilde{R}_{j} \right)=\frac{D\left( \tilde{R}_{i}, \tilde{R}_{j} \right)}{D^{*}\left( \tilde{R}_{p}, \tilde{R}_{q} \right)}$ (7)

where $D\left( \tilde{R}_{i}, \tilde{R}_{j} \right)$ is the normalized distance measure between each pair of experts $\tilde{R}_{i}\mathrm{and}\tilde{R}_{j}$ based on the weighted Hamming distance $d_{H}\left( \tilde{R}_{i},\tilde{R}_{j} \right)$ and the distance $d_{inf}\left( \tilde{R}_{i},\tilde{R}_{j} \right)$ as follows:

$$D\left( \tilde{R}_{i}, \tilde{R}_{j} \right)=\frac{1}{2}\left( d_{H}\left( \tilde{R}_{i},\tilde{R}_{j} \right)+d_{inf}\left( \tilde{R}_{i},\tilde{R}_{j} \right) \right) (8)$$

and $d_{H}\left( \tilde{R}_{i},\tilde{R}_{j} \right)$ and $d_{inf}\left( \tilde{R}_{i},\tilde{R}_{j} \right)$ are calculated between any two fuzzy numbers $\tilde{R}_{i}=\left( a_{i}, b_{i}, c_{i} \right)=a_{1},b_{1},c_{1} and$ $\tilde{R}_{j}=\left( a_{j}, b_{j}, c_{j} \right)=a_{2},b_{2},c_{2}$ as follows:

$$d_{H}\left( \tilde{R}_{i},\tilde{R}_{j} \right)= \sum_{m=1}^{3} |a_{m}-b_{m}| (9)$$

$$d_{inf}\left( \tilde{R}_{i},\tilde{R}_{j} \right)= inf(d\left( a, b \right)), a \in\tilde{R}_{i}, b\in\tilde{R}_{j} (10)$$

1. Calculating the consistency degree between each pair of experts. Given $\beta\epsilon[0,1]$, let $\beta=0.5$ calculate the consistency degree $r\left( \tilde{R}_{i}, \tilde{R}_{j} \right)$ between each pair of medical professionals by:

$$r\left( \tilde{R}_{i}, \tilde{R}_{j} \right)=\beta S_{w}\left( \tilde{R}_{i}, \tilde{R}_{j} \right)+\left( 1-\beta\right)\left( 1-d\left( \tilde{R}_{i}, \tilde{R}_{j} \right) \right) (11)$$

where $\beta$ is the weight of $S_{w}\left( \tilde{R}_{i}, \tilde{R}_{j} \right)$, which reflects the relative importance degree between the similarity and the distance to the decision-maker. As a result, $\left( 1-\beta\right)$ is the weight associated with the distance, $d\left( \tilde{R}_{i}, \tilde{R}_{j} \right)$.

1. Selecting the degree of importance $e_{i}$ for each expert. In practice, group decision-making is highly influenced by the degrees of importance of the domain participants [8-10]. The degree of importance is defined according to the years of experience and knowledge reported by each participant. So, for the aggregation method to be effective, the relative years of experience weight of each participant is defined as the ratio of each medical professional over the total years of experience in the panel. Without any loss of generality, let the degree of importance *i*th expert be $e_{i}\left( 0 \leq e_{i}\leq1 \right)$ and

$$\sum_{i=1}^{n} e_{i}=1 (12)$$

then, we calculate the weighted consistency degree $C\left( E_{i} \right)$ for each expert $E_{i}$ as

$$C\left( E_{i} \right)=\sum_{j=1}^{n} r(\tilde{R}_{i}, \tilde{R}_{j})e_{i} (13)$$

1. Computing the aggregation weight of each expert $w\left( E_{i} \right)$ by equation 14

$w\left( E_{i} \right)=\frac{C\left( E_{i} \right)}{\sum_{i=1}^{n} C\left( E_{i} \right)} (14)$

1. Aggregating each fuzzy opinion for each factor *O* defined in the Mathematical Expression into a matrix which contains the fuzzy group opinion as:

$$\tilde{R}_{O}=\sum_{i=1}^{n} w\left( E_{i} \right)\odot\tilde{R}_{i} => \binom{\begin{aligned} \left[ w\left( E_{i} \right)\times a_{1}+\ldots+w\left( E_{n} \right)\times a_{n} \right], \\ \left[ w\left( E_{i} \right)\times b_{1}+\ldots+w\left( E_{n} \right)\times b_{n} \right], \end{aligned}}{\left[ w\left( E_{i} \right)\times c_{1}+\ldots+w\left( E_{n} \right)\times c_{n} \right]} =\left( a_{k},b_{k},c_{k} \right) \left( 15 \right)$$

where $k = 1, \ldots, K$, and $\odot$ is the fuzzy multiplication operator.

1. Defuzzification. When solving a decision problem via a fuzzy approach, the output is preferred to be a crisp value (i.e., a single number) and not a fuzzy set. To achieve that, the fuzzy set we obtained in *equation 15* should be converted into a single numerical value. One of the most common defuzzification methods is the center of gravity method (COG), which returns the weighted average of the membership function of the given fuzzy set obtained in step 7. The equation is as follows, $R_{i}= (a_{k}, b_{k}, c_{k})$ is defuzzified by:

$$S_{k}=\frac{\left( a_{k}, b_{k}, c_{k} \right)}{3}, k = 1,\ldots, K (16)$$

2. Threshold settings. The cut-off point is determined using the median of the indicators in each factor [11]. If the group agreement for the indicators does not meet the cut-off, then a new round is established (see equation 16 and 17).

$$If S_{k}<the set median threshold, discard variable k (16)$$

$$If S_{k}\geq the set median threshold, retain the variable k (17)$$

*Text mining*: Sentiment analysis is a text analysis method that detects polarity (e.g., a positive or negative opinion) within a given comment. Sentiment analysis is an active area of study in the field of natural language processing that aims to measure the opinions, sentiments, and evaluations of participants via the computational treatment of subjectivity in text. We employ a very popular and powerful tool developed by [12] called VADER (Valence Aware Dictionary for Sentiment Reasoning) for sentimental analysis of expert opinions. VADER is used for text sentiment analysis that is sensitive to both polarity (i.e., positive, or negative comments) and intensity (i.e., strength) of emotion. We aggregate all comments into text corpus. We clean the data from stop words and lemmatize it before inputting into VADER. A dictionary is created that maps lexical features to emotion intensities known as sentiment scores. The sentiment score of a text can be obtained by summing up the intensity of each word in the text. The results produce the sentiment intensity and understanding can be extended with topics analysis that gives us insights into why medical professionals rated certain items high or low. The proposed approach is implemented in Python environment. The detailed steps above can be used by any hospital data scientist/analyst to recreate the study. The GitHub link of our source code can be found by clicking: [MOFD method](https://github.com/Dergel0806/Consistency-Aggregation-Method) and [FDM method](https://github.com/Dergel0806/Traditional-Delphi-Method).

**Results**


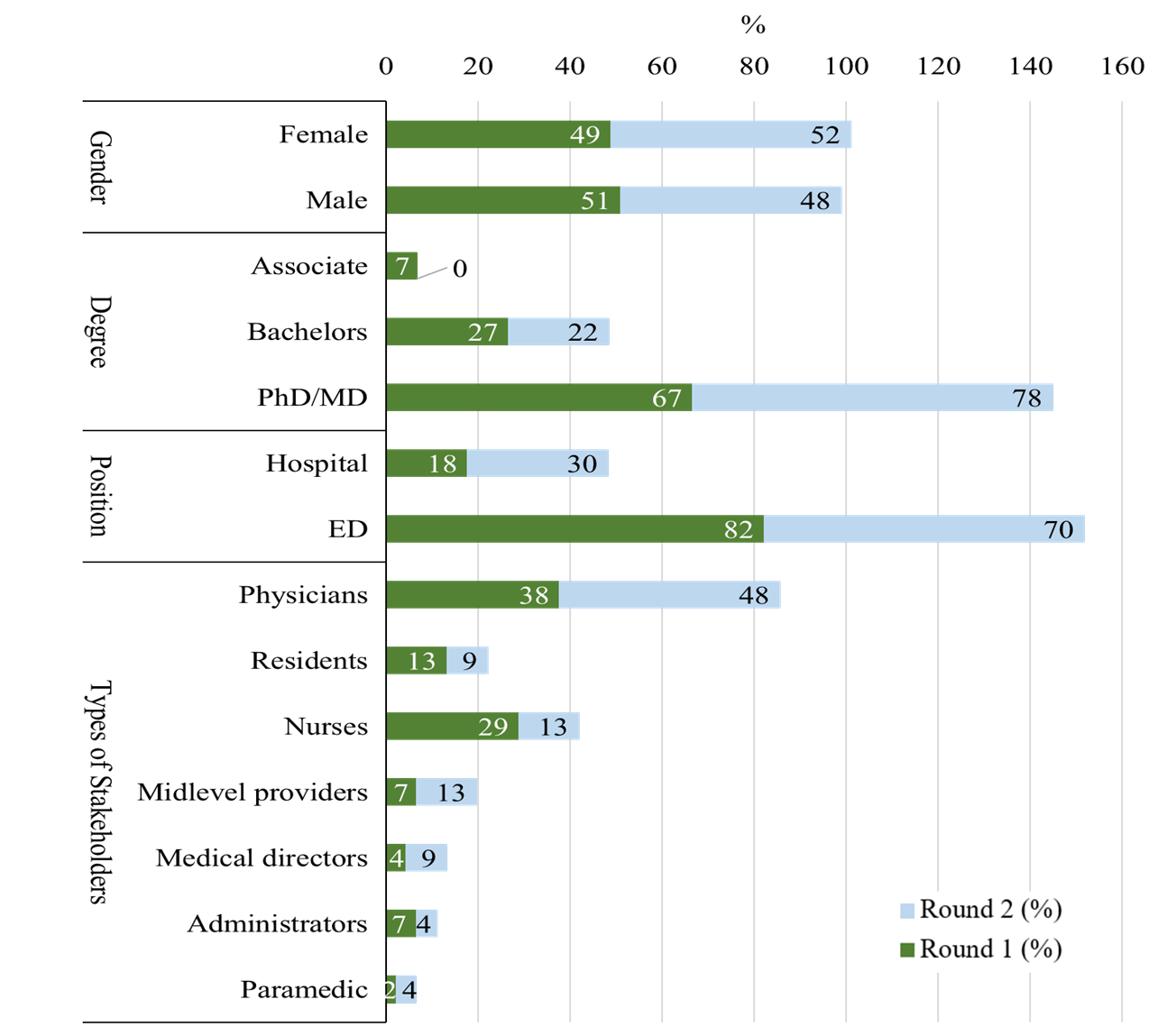


**S2 Fig. Demographic characteristics of experts involved in the two-round MOFD study (more details can be found in S4 Table)**

| **S4 Table. Demographic characteristics of experts involved in the two-round MOFD study** | | | | | |
| --- | --- | --- | --- | --- | --- |
| Characteristics | Categories | First Round | | Second Round | |
|  |  | N | P (%) | N | P (%) |
| Gender | Female | 22 | 48.89 | 12 | 26.67 |
|  | Male | 23 | 51.11 | 11 | 24.44 |
| Disaster experience | Yes (e.g., $H_{1}N_{1}$ influenza, SARS, mass casualty events) | 45 | 100 | 23 | 100 |
|  | No experience | 0 | 0 | 0 | 0 |
| Degree | Associate degree | 3 | 6.67 | 0 | 0 |
|  | Bachelor’s degree | 12 | 26.67 | 5 | 11.11 |
|  | PhD/MD | 30 | 66.67 | 18 | 40.00 |
| Current Place of Employment | Hospital | 8 | 17.78 | 7 | 15.56 |
|  | Emergency Department | 37 | 82.22 | 16 | 35.56 |
|  | None | 0 | 0 | 0 | 0 |
| Types of Stakeholders | Physicians | 17 | 37.78 | 11 | 47.82 |
|  | Resident physicians | 6 | 13.33 | 2 | 8.69 |
|  | Registered / Clinical nurses | 13 | 28.89 | 3 | 13.04 |
|  | Midlevel providers | 3 | 6.67 | 3 | 13.04 |
|  | Medical directors | 2 | 4.44 | 2 | 8.69 |
|  | Administrators | 3 | 6.67 | 1 | 4.35 |
|  | Paramedic | 1 | 2.22 | 1 | 4.35 |
| Total | | 45 | 100.00 | 23 | 51.11 |

Note: **N**: Total number; **P**: Percentage; **SARS**: Severe acute respiratory syndrome; **PhD**: Doctor of Philosophy; **MD**: Medical Doctor

| **S5 Table. Weights (degrees of importance) of the years of experience of expert** | | | | |
| --- | --- | --- | --- | --- |
| Job | Gender | Years of experience | Expert’s Weights | |
|  |  |  | $e_{i}$ | % |
| Physician | Male | 7 | e1 | 0.023 |
| Physician | Male | 10 | e2 | 0.033 |
| Physician | Male | 3 | e3 | 0.010 |
| Physician | Male | 7 | e4 | 0.023 |
| Physician | Male | 8 | e5 | 0.026 |
| Physician | Male | 7 | e6 | 0.023 |
| Physician | Male | 9 | e7 | 0.030 |
| Physician | Female | 7 | e8 | 0.023 |
| Physician | Male | 5 | e9 | 0.016 |
| Physician | Female | 7 | e10 | 0.023 |
| Physician | Female | 8 | e11 | 0.026 |
| Physician | Male | 7 | e12 | 0.023 |
| Physician | Male | 8 | e13 | 0.026 |
| Physician | Female | 7 | e14 | 0.023 |
| Physician | Male | 5 | e15 | 0.016 |
| Physician | Male | 4 | e16 | 0.013 |
| Physician | Male | 5 | e17 | 0.016 |
| Resident Physician | Female | 6 | e18 | 0.020 |
| Resident Physician | Female | 3 | e19 | 0.010 |
| Resident Physician | Male | 3 | e20 | 0.010 |
| Resident Physician | Female | 3 | e21 | 0.010 |
| Resident Physician | Female | 3 | e22 | 0.010 |
| Resident Physician | Female | 3 | e23 | 0.010 |
| Registered Nurse | Male | 7 | e24 | 0.023 |
| Registered Nurse | Female | 7 | e25 | 0.023 |
| Registered Nurse | Male | 9 | e26 | 0.030 |
| Registered Nurse | Female | 8 | e27 | 0.026 |
| Registered Nurse | Female | 10 | e28 | 0.033 |
| Registered Nurse | Female | 7 | e29 | 0.023 |
| Registered Nurse | Male | 9 | e30 | 0.030 |
| Registered Nurse | Female | 9 | e31 | 0.030 |
| Registered Nurse | Female | 11 | e32 | 0.036 |
| Registered Nurse | Male | 10 | e33 | 0.033 |
| Registered Nurse | Female | 9 | e34 | 0.030 |
| Registered Nurse | Male | 7 | e35 | 0.023 |
| Clinical nurse specialist | Male | 8 | e36 | 0.026 |
| Midlevel provider | Female | 8 | e37 | 0.026 |
| Midlevel provider | Female | 7 | e38 | 0.023 |
| Midlevel provider | Female | 3 | e39 | 0.010 |
| Medical director | Male | 7 | e40 | 0.023 |
| Medical director | Male | 9 | e41 | 0.030 |
| Administrator | Female | 3 | e42 | 0.010 |
| Administrator | Female | 7 | e43 | 0.023 |
| Administrator | Female | 8 | e44 | 0.026 |
| Paramedic | Male | 6 | e45 | 0.020 |

| **S6 Table. First-round results of DM** | | | | | |
| --- | --- | --- | --- | --- | --- |
| Healthcare performance factors | Indicators | Median | IQR | Freq. | Consensus |
| Capacity | ED beds | 1.0 | 1.0 | 0.044 | FALSE |
|  | ICU beds | 1.0 | 1.0 | 0.067 | FALSE |
|  | Physician staffing | 2.0 | 3.0 | 0.356 | FALSE |
|  | Midlevel provider staffing | 3.0 | 2.0 | 0.333 | FALSE |
|  | Nurse staffing | 2.0 | 1.0 | 0.133 | FALSE |
|  | Patient acuity level | 4.0* | 2.0 | 0.711 | TRUE |
|  | Physician staffing per patient seen | 4.0* | 2.0 | 0.733 | TRUE |
|  | Nurse staffing per patient seen | 5.0* | 1.0 | 0.800 | TRUE |
|  | Backup physician | 3.0 | 1.0 | 0.422 | FALSE |
|  | Backup nurse | 4.0* | 2.0 | 0.578 | TRUE |
|  | Patient care compromised | 4.0* | 1.0 | 0.8 | TRUE |
|  | Medical support personnel | 2.0 | 2.0 | 0.378 | FALSE |
| Temporal | High acuity | 3.0 | 2.0 | 0.378 | FALSE |
|  | Low acuity | 1.0 | 1.0 | 0.111 | FALSE |
|  | Admit ED LOS < 6 hrs. | 1.0 | 1.0 | 0.089 | FALSE |
|  | Discharge ED LOS < 4 hrs. | 1.0 | 1.0 | 0.044 | FALSE |
|  | Time to triage | 3.0 | 1.0 | 0.244 | FALSE |
|  | Time to start of treatment | 3.0 | 1.0 | 0.222 | FALSE |
|  | Time to ED bed | 3.0 | 2.0 | 0.244 | FALSE |
|  | Time to treatment condition | 2.0 | 1.0 | 0.133 | FALSE |
| Quality | Employee fatigue | 5.0* | 1.0 | 1.000 | TRUE |
|  | Employee satisfaction | 1.0 | 1.0 | 0.044 | FALSE |
|  | Medical errors | 4.0* | 2.0 | 0.733 | TRUE |
| Outcomes | Patients hospitalized | 3.0 | 1.0 | 0.356 | FALSE |
|  | Patient transfers | 2.0 | 2.0 | 0.422 | FALSE |
| Financial expenditures | Increase diagnostic test | 4.0* | 1.0 | 0.600 | TRUE |
|  | Increase ED treatment | 3.0 | 2.0 | 0.444 | FALSE |
|  | Increase ED revenue | 3.0 | 0.0 | 0.222 | FALSE |
|  | Increase in non-labor cost | 4.0* | 1.0 | 0.667 | TRUE |

The values with (*) show consensus based on group opinions for each metric (Median ≥ 4)

*Note – ED: Emergency Department; LOS: Length of stay; DM: Delphi Method; IQR: Interquartile range

| S7 Table. MOFD results for normal operating conditions | | | | |
| --- | --- | --- | --- | --- |
| Healthcare performance factors | Indicators | Avg. of fuzzy numbers | Consensus (threshold > 36.81) | Rank |
| Capacity | ED beds | 0.355 | 35.872 | 20 |
|  | ICU beds | 0.353 | 33.697 | 28 |
|  | Physician staffing | 0.467 | 36.056 | 19 |
|  | Midlevel provider staffing | 0.469 | 36.852* | 14 |
|  | Nurse staffing | 0.402 | 33.902 | 26 |
|  | Patient acuity level | 0.195 | 34.931 | 22 |
|  | Physician staffing per patient seen | 0.278 | 33.909 | 25 |
|  | Nurse staffing per patient seen | 0.201 | 37.307* | 12 |
|  | Backup physician | 0.272 | 34.278 | 23 |
|  | Backup nurse | 0.232 | 35.597 | 21 |
|  | Patient care compromised | 0.255 | 36.515 | 17 |
|  | Medical support personnel | 0.316 | 32.965 | 29 |
| Temporal | High acuity | 0.303 | 33.941 | 24 |
|  | Low acuity < 60 mins | 0.345 | 33.746 | 27 |
|  | Admit ED LOS < 6 hrs | 0.215 | 36.654 | 16 |
|  | Discharge ED LOS < 4 hrs | 0.284 | 37.104* | 13 |
|  | Time to triage | 0.432 | 42.597* | 8 |
|  | Time to start of treatment | 0.414 | 43.545* | 4 |
|  | Time to ED bed | 0.389 | 40.827* | 9 |
|  | Time to treatment condition | 0.369 | 42.772* | 6 |
| Quality | Employee fatigue | 0.431 | 36.097 | 18 |
|  | Employee satisfaction | 0.433 | 36.810* | 15 |
|  | Medical errors | 0.349 | 42.896* | 5 |
| Outcomes | Patients hospitalized | 0.355 | 52.048* | 3 |
|  | Patient transfers | 0.223 | 42.602* | 7 |
| Financial expenditures | Increase diagnostic test | 0.411 | 40.304* | 11 |
|  | Increase ED treatment | 0.404 | 40.654* | 10 |
|  | Increase ED revenue | 0.384 | 53.416* | 2 |
|  | Increase in non-labor cost | 0.385 | 53.512* | 1 |

| **S8 Table. Bayesian Change Point Analysis & T-test for Indicator Validation** | | | | | |
| --- | --- | --- | --- | --- | --- |
| Indicators | Year 2019 | Year 2020 | Paired T-test | | Observation |
| Nurse staffing | 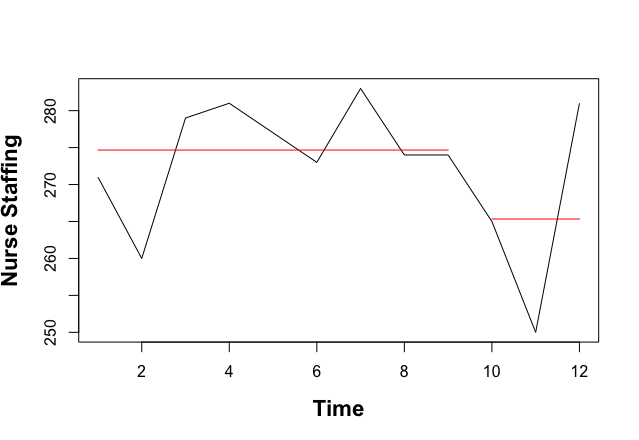 | 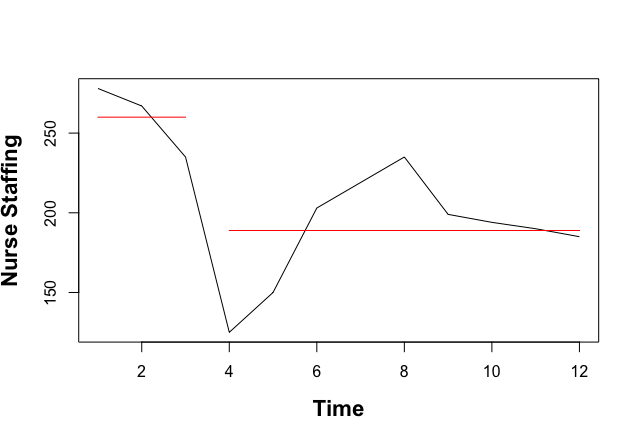 | P_value_ = 0.000^a^ | | There is a change in the number of nurses in the ED in both 2019 and 2020. |
| Midlevel provider staffing | 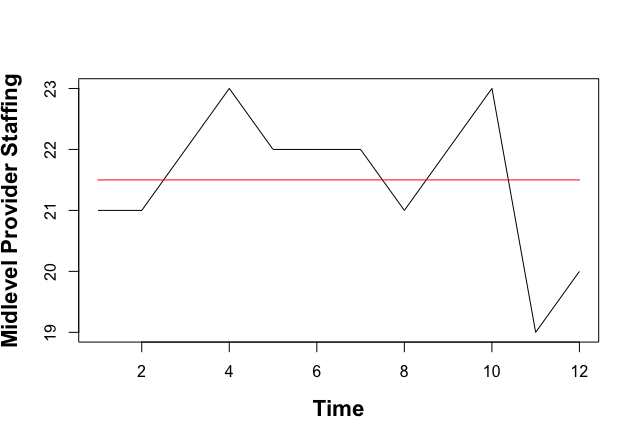 | 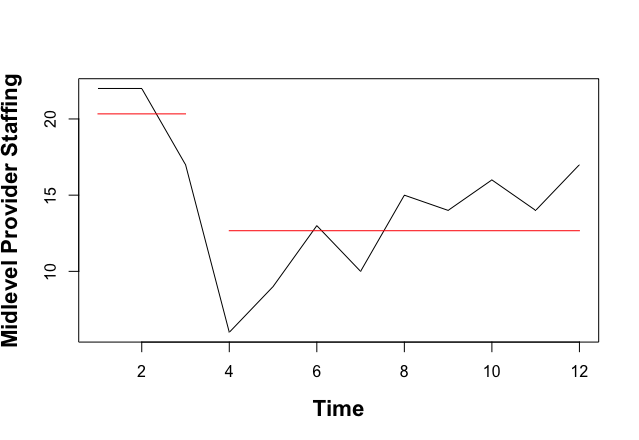 | P_value_ = 0.000^a^ | | In 2019, there is no change observed in the number of Midlevel providers in the ED, but in April 2020, we can see a change in the staffing level of midlevel providers. |
| ICU Bed | 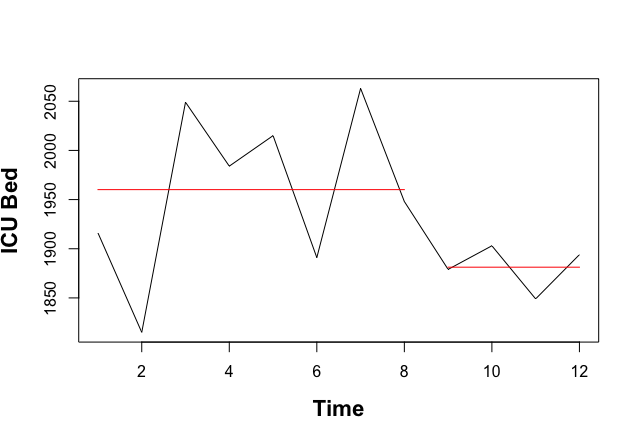 | 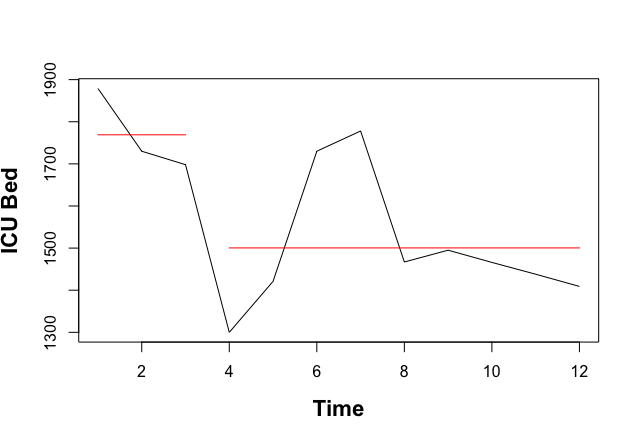 | P_value_ = 0.000^a^ | | In August 2019, there is a reduction in the number of ICU beds used, and in 2020, the change is observed in March. |
| Time to start of treatment | 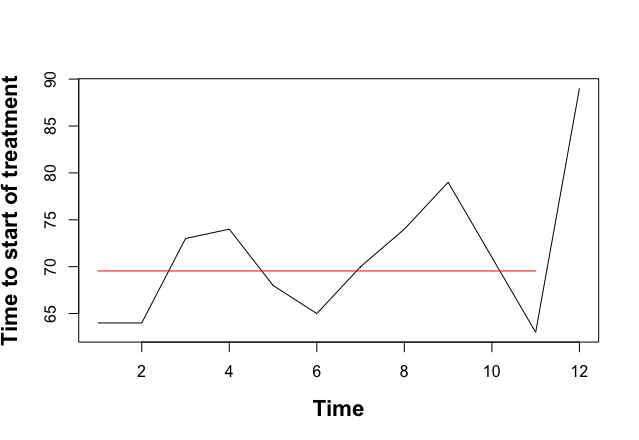 | 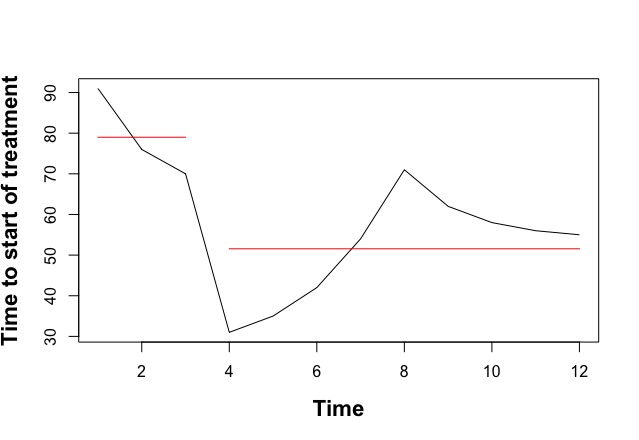 | P_value_ =0.047^a^ | | There is an observed change in the average time to start of treatment for patients who visit the ED in November 2019. This change can be observed in March of 2020. |
| Time to triage | 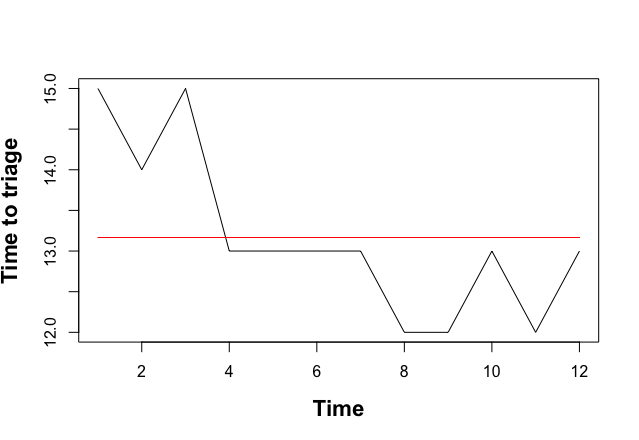 | 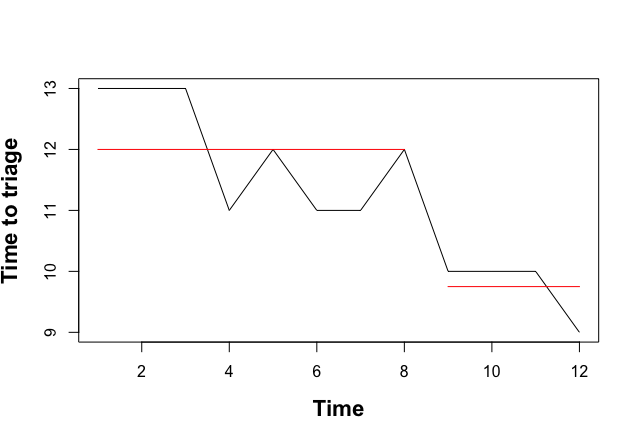 | P_value_ = 0.000^a^ | | No change was observed in 2019, but a change was observed in August 2020. |
| Admit ED Length of Stay | 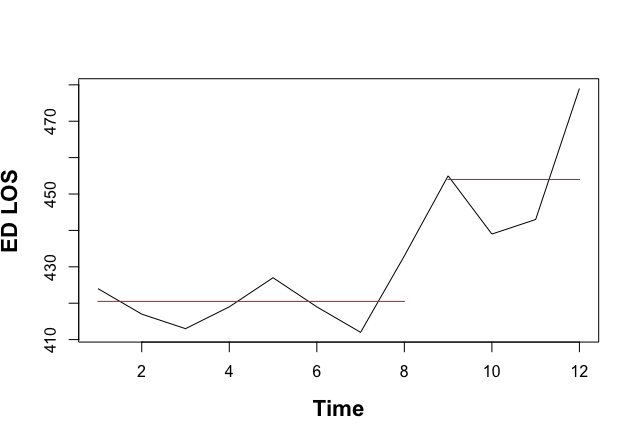 | 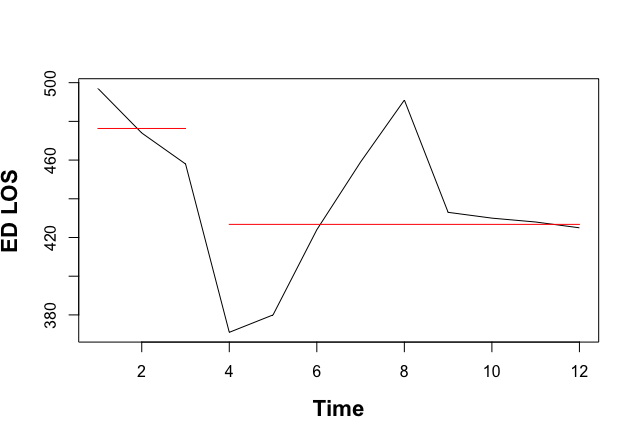 | | P_value_ = 0.587 | There is an observed change in the average ED Length of stay in August 2019 and March 2020. |

^a^Statistically significant at 5% significance level

Additional References

1. Mengual-Andrés S, Roig-Vila R, Mira JB. Delphi study for the design and validation of a questionnaire about digital competences in higher education. International Journal of Educational Technology in Higher Education. 2016;13(1):12.

2. Ma Z, Shao C, Ma S, Ye Z. Constructing road safety performance indicators using fuzzy delphi method and grey delphi method. Expert Systems with Applications. 2011;38(3):1509-14.

3. Chang P-C, Wang Y-W. Fuzzy Delphi and back-propagation model for sales forecasting in PCB industry. Expert systems with applications. 2006;30(4):715-26.

4. Chang P-T, Huang L-C, Lin H-J. The fuzzy Delphi method via fuzzy statistics and membership function fitting and an application to the human resources. Fuzzy sets and systems. 2000;112(3):511-20.

5. Lu C, Lan J, Wang Z. Aggregation of fuzzy opinions under group decision-making based on similarity and distance. Journal of Systems Science and Complexity. 2006;19(1):63-71.

6. Park JW, Yun YS, Kang KH. The mean value and variance of one-sided fuzzy sets. Journal of the Chungcheong Mathematical Society. 2010;23(3):511-21.

7. Guha D, Chakraborty D. A new approach to fuzzy distance measure and similarity measure between two generalized fuzzy numbers. Applied Soft Computing. 2010;10(1):90-9.

8. Foth T, Efstathiou N, Vanderspank-Wright B, Ufholz L-A, Dütthorn N, Zimansky M, et al. The use of Delphi and Nominal Group Technique in nursing education: a review. International Journal of Nursing Studies. 2016;60:112-20.

9. DiCenso A, Guyatt G, Ciliska D. Evidence-based nursing: A guide to clinical practice: Elsevier Health Sciences; 2005.

10. Haynes RB, Sackett DL, Richardson WS, Rosenberg W, Langley GR. Evidence-based medicine: How to practice & teach EBM. Canadian Medical Association Journal. 1997;157(6):788.

11. Madsen MM, Eiset AH, Mackenhauer J, Odby A, Christiansen CF, Kurland L, et al. Selection of quality indicators for hospital-based emergency care in Denmark, informed by a modified-Delphi process. Scandinavian journal of trauma, resuscitation and emergency medicine. 2016;24(1):11.

12. Gilbert C, Hutto E, editors. Vader: A parsimonious rule-based model for sentiment analysis of social media text. Eighth International Conference on Weblogs and Social Media (ICWSM-14) Available at (20/04/16) <http://comp> social gatech edu/papers/icwsm14 vader hutto pdf; 2014.
