# Supplementary material for: Identifying indicators influencing emergency department performance during a medical surge: A consensus-based modified fuzzy Delphi approach
Source: PLoS One. 2022 Apr 21;17(4):e0265101. doi: 10.1371/journal.pone.0265101 (PMC9022798; doi:10.1371/journal.pone.0265101)
Supplement: Attachment — Submitted filename: Response to Reviewers.docx [file pone.0265101.s002.docx]

Response to Reviewers: PONE-D-21-39883

Dear editor and reviewers,

Thank you for giving us the opportunity to submit a revised draft of our manuscript titled “Identifying Indicators Influencing Emergency Department Performance during a Medical Surge: A Consensus-Based Modified Fuzzy Delphi Approach”. We appreciate the time and effort that you and the reviewers have dedicated to providing your valuable feedback on our manuscript. We are grateful to the reviewers for their insightful comments. We have been able to incorporate changes to reflect all of the suggestions. Also, we have highlighted the changes within the manuscript.

Here is a point-by-point response to the editor and reviewers’ comments and concerns.

Journal Requirements:

When submitting your revision, we need you to address these additional requirements.

1. Please ensure that your manuscript meets PLOS ONE's style requirements, including those for file naming. The PLOS ONE style templates can be found at 
<https://journals.plos.org/plosone/s/file?id=wjVg/PLOSOne_formatting_sample_main_body.pdf> and 
<https://journals.plos.org/plosone/s/file?id=ba62/PLOSOne_formatting_sample_title_authors_affiliations.pdf>

Additional Editor Comments:

The manuscript has been reviewed by two experts in the area. Both of the referees appreciate the value of the paper. However, Reviewer 2 has some concerns about the organization of the manuscript and the writing. I have gone through the manuscript and agree with the referees. This study on ED performance is important and has value. I also agree with Reviewer 2 that the organization of the work shall be strengthened to reflect the contributions and key messages of the work (report of results / development of the methodology).

In addition, I have one minor suggestion. The majority of the references used in this study are not recent. To review the state of the art and show the timeliness of the study, the authors may include some more recent relevant studies to interest the reader. I realize some of the recent related studies, e.g.,:
Ansah, J. P., Ahmad, S., Lee, L. H., Shen, Y., Ong, M. E. H., Matchar, D. B., & Schoenenberger, L. (2021). Modeling Emergency Department crowding: Restoring the balance between demand for and supply of emergency medicine. Plos one, 16(1), e0244097.
Barak-Corren, Y., Chaudhari, P., Perniciaro, J., Waltzman, M., Fine, A. M., & Reis, B. Y. (2021). Prediction across healthcare settings: a case study in predicting emergency department disposition. NPJ digital medicine, 4(1), 1-7
Benevento, E., Aloini, D., & Squicciarini, N. (2021). Towards a real-time prediction of waiting times in emergency departments: A comparative analysis of machine learning techniques. International Journal of Forecasting.
Kuo, Y. H., Chan, N. B., Leung, J. M., Meng, H., So, A. M. C., Tsoi, K. K., & Graham, C. A. (2020). An integrated approach of machine learning and systems thinking for waiting time prediction in an emergency department. International journal of medical informatics, 139, 104143.
Kuo, Y. H., Rado, O., Lupia, B., Leung, J. M., & Graham, C. A. (2016). Improving the efficiency of a hospital emergency department: a simulation study with indirectly imputed service-time distributions. Flexible Services and Manufacturing Journal, 28(1-2), 120-147.
Tang, K. J. W., Ang, C. K. E., Constantinides, T., Rajinikanth, V., Acharya, U. R., & Cheong, K. H. (2021). Artificial intelligence and machine learning in emergency medicine. Biocybernetics and Biomedical Engineering, 41(1), 156-172.
Vanbrabant, L., Braekers, K., Ramaekers, K., & Van Nieuwenhuyse, I. (2019). Simulation of emergency department operations: A comprehensive review of KPIs and operational improvements. Computers & Industrial Engineering, 131, 356-381.

We thank the editor for the valuable feedback. We have revised and updated the manuscript to reflect the contributions and key messages. The citation and reference list has been updated to capture the suggested references provided by the editor. Lastly, the manuscript has been properly formatted to meet the journal’s requirements.

Now the manuscript reads at the reference section:

“[2] Ansah, J. P., Ahmad, S., Lee, L. H., Shen, Y., Ong, M. E. H., Matchar, D. B., & Schoenenberger, L. (2021). Modeling Emergency Department crowding: Restoring the balance between demand for and supply of emergency medicine. Plos one, 16(1), e0244097.

[17] Benevento, E., Aloini, D., & Squicciarini, N. (2021). Towards a real-time prediction of waiting times in emergency departments: A comparative analysis of machine learning techniques. International Journal of Forecasting.

[11] Kuo, Y. H., Chan, N. B., Leung, J. M., Meng, H., So, A. M. C., Tsoi, K. K., & Graham, C. A. (2020). An integrated approach of machine learning and systems thinking for waiting time prediction in an emergency department. International journal of medical informatics, 139, 104143.

[12] Kuo, Y. H., Rado, O., Lupia, B., Leung, J. M., & Graham, C. A. (2016). Improving the efficiency of a hospital emergency department: a simulation study with indirectly imputed service-time distributions. Flexible Services and Manufacturing Journal, 28(1-2), 120-147.

[10] Tang, K. J. W., Ang, C. K. E., Constantinides, T., Rajinikanth, V., Acharya, U. R., & Cheong, K. H. (2021). Artificial intelligence and machine learning in emergency medicine. Biocybernetics and Biomedical Engineering, 41(1), 156-172.

[21] Vanbrabant, L., Braekers, K., Ramaekers, K., & Van Nieuwenhuyse, I. (2019). Simulation of emergency department operations: A comprehensive review of KPIs and operational improvements. Computers & Industrial Engineering, 131, 356-381.”

Reviewer #1: Thanks for the opportunity. This was a well-written and easy-to-follow article with a significant scientific value. Numerous methods have been described in the literature to identify Emergency Department performance throughout years. These methods are useful as a guide to enhance quality of care in workplace productivity in Emergency departments. This is a well-written and well-designed article on indicators of Emergency department performance. Particularly in the COVID-19 era, we hope that number of such researches increase.
Best regards.

We thank the reviewer for their valuable comments on the manuscript.

Reviewer #2: The overall message of this project is valuable (identifying perfomance indicators for ED's during a surge) and is highly relevant given the pandemic, however it requires massive editing. As written, it can be difficult to tell if it is a traditional manuscript reporting results or a methodology paper. Some of this has to do with verb tense agreement (recommend using simple past tense throughout). It also requres an overall edit for grammar, targeting both verb tense correction and elimination of sentence fragments.
I would find it acceptable if the authors described the problem and said that they used a fuzzy Delphi approach as their methodology. Pages 4-8 can be deleted or significantly shortened. Similarly, pages 9-12 can be significantly condensed to only give key details on how the process was implemented. Pages 13-22 provide more substantial content (results) but could still be edited to minimize overly detailed references to methods. A major overhaul in these sections could significantly improve the clarity and readability of the manuscript.

We thank the reviewer for their valuable comments on the manuscript. The manuscript has been improved to explain the key details of the work in the introduction, methodology, and result sections. Also, the suggested sections by the reviewer have been significantly shortened to improve the clarity and readability of the manuscript.
